# Whole Ovary Cryopreservation and Transplantation: A Systematic Review of Challenges and Research Developments in Animal Experiments and Humans

**DOI:** 10.3390/jcm9103196

**Published:** 2020-10-02

**Authors:** Camille Hossay, Jacques Donnez, Marie-Madeleine Dolmans

**Affiliations:** 1 Pôle de Recherche en Gynécologie, Institut de Recherche Expérimentale et Clinique, Université Catholique de Louvain, 1200 Brussels, Belgium; camille.hossay@uclouvain.be; 2 Society for Research into Infertility, 1150 Brussels, Belgium; jacques.donnez@gmail.com; 3 Gynecology Department, Cliniques Universitaires Saint-Luc, 1200 Brussels, Belgium

**Keywords:** whole ovary, cryopreservation, vascular anastomosis, microsurgery

## Abstract

Ovarian tissue cryopreservation and transplantation is the only fertility preservation option that enables both restoration of fertility and resumption of ovarian endocrine function, avoiding the morbidity associated with premature menopause. It is also the only technique available to prepubertal patients and those whose treatment cannot be delayed for life-threatening reasons. Ovarian tissue cryopreservation can be carried out in two different ways, either as ovarian cortical fragments or as a whole organ with its vascular pedicle. Although use of cortical strips is the only procedure that has been approved by the American Society for Reproductive Medicine, it is fraught with drawbacks, the major one being serious follicle loss occurring after avascular transplantation due to prolonged warm ischemia. Whole ovary cryopreservation involves vascular transplantation, which could theoretically counteract the latter phenomenon and markedly improve follicle survival. In theory, this technique should maintain endocrine and reproductive functions much longer than grafting of ovarian cortical fragments. However, this procedure includes a number of critical steps related to (A) the level of surgical expertise required to accomplish retrieval of a whole ovary with its vascular pedicle, (B) the choice of cryopreservation technique for freezing of the intact organ, and (C) successful execution of functional vascular reanastomosis upon thawing. The aim of this systematic review is to shed light on these challenges and summarize solutions that have been proposed so far in animal experiments and humans in the field of whole ovary cryopreservation and transplantation.

## 1. Introduction

Increased incidence of cancer in females <45 years of age, along with major advances in cancer therapy over recent decades, has resulted in a growing population of girls and women surviving cancer [1]. Some of these cancer survivors will subsequently face premature ovarian insufficiency (POI) because of the gonadotoxic effect of certain cancer treatments, like chemotherapy, radiotherapy and surgery [2,3,4,5,6,7,8]. Various autoimmune diseases and benign hematological conditions may also require gonadotoxic therapy, leading to iatrogenic ovarian failure [6,9]. Even benign ovarian pathologies could give rise to the same unfortunate outcome when multiple ovarian cystectomies are performed [10,11]. In some cases, the pathology itself may cause ovarian insufficiency, as in several genetic predispositions (e.g., Turner syndrome) [12]. This loss of ovarian function results in cessation of sex steroid secretion, which prevents pubertal development in prepubertal girls and causes early menopause and ensuing infertility in women of reproductive age [13]. It also has significant systemic side effects, with an increased risk of cardiovascular disease, osteoporosis, earlier mortality, obesity, sexual dysfunction, depression, dementia and cognitive decline [14]. POI clearly has a severe impact on physical, mental and social well-being.

With quality of life steadily gaining greater attention, advances in reproductive medicine have led to the development and increased use of multiple fertility preservation techniques. Embryo cryopreservation, mature oocyte cryopreservation and ovarian tissue cryopreservation are currently the only methods of fertility preservation endorsed by the American Society for Reproductive Medicine [15]. Different parameters must be considered when selecting the appropriate approach: age, type of condition, treatment required, timing of gonadotoxic therapy and partner status [6,16]. Ovarian tissue cryopreservation and subsequent transplantation is the sole option that enables both restoration of fertility and resumption of ovarian endocrine function, avoiding the morbidity associated with POI [6]. It is also the only technique available to prepubertal patients and those whose treatment cannot be delayed for life-threatening reasons, ruling out ovarian stimulation required for oocyte pick-up and oocyte/embryo vitrification [6,9,16,17].

Ovarian tissue cryopreservation can be achieved in two different ways. It can be done in the form of ovarian cortical fragments that are grafted in an avascular manner after thawing, or the whole organ can be frozen with its vascular pedicle and reimplanted by means of vascular anastomosis once thawed [18]. Ovarian tissue cryopreservation and transplantation in the form of cortical strips is now a widely used technique, yielding resumption of endocrine ovarian function in 95% of women, pregnancy rates of 30–50%, and over 200 live births to date [6,19,20,21,22]. Nevertheless, transplanted fragments contain only a fraction of a patient’s ovarian reserve and therefore provide a relatively brief fertile window before complete depletion of oocytes contained within the graft [6,23,24,25]. The issue of graft longevity is exacerbated by the fact that up to 80% of the initial follicle stockpile is lost in the early postgrafting period [26,27,28,29,30]. This massive follicle loss is mainly due to prolonged warm ischemia that the tissue sustains following avascular transplantation, during which time neovascularization is established [21,31,32,33].

Whole ovary cryopreservation and transplantation may circumvent the dramatic follicle loss and subsequently shortened graft life span and function observed when using cortical strips. Indeed, transplantation of a frozen–thawed intact ovary with its vascular pedicle may theoretically provide immediate reperfusion of the organ through successful vascular reanastomosis, which reduces warm ischemia from several days to just a few hours, in turn markedly improving follicle survival. Furthermore, freezing of a whole ovary involves preservation of all the primordial follicles inside the ovary, making this approach even more attractive. In theory, this technique should maintain endocrine and reproductive functions much longer than grafting of ovarian cortical fragments [18,34,35,36,37]. It should also be more favorable for patients of reproductive age who have a low ovarian reserve [38]. The aim of this systematic review is to summarize the challenges faced and research advances made in animal experiments and humans over the past 20 years in the field of whole ovary cryopreservation and transplantation.

## 2. Methodology

**Figure 1:**
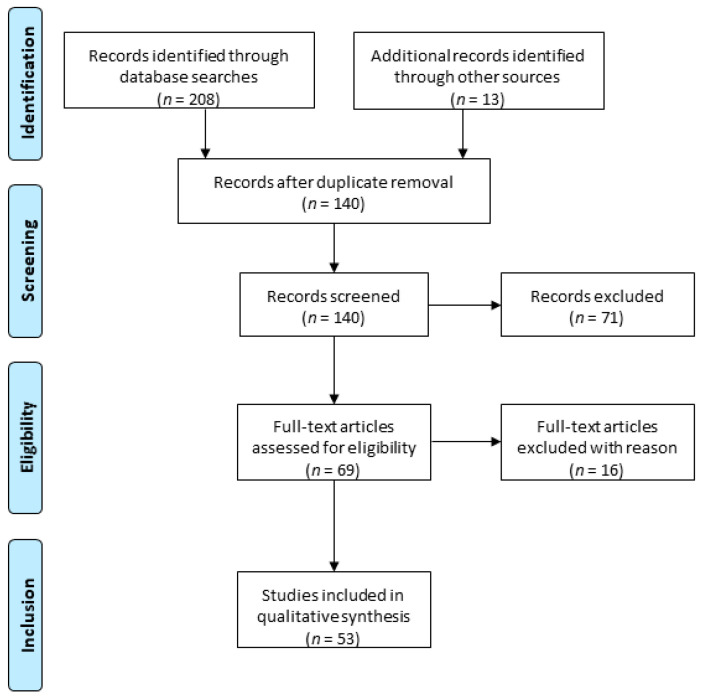
Preferred Reporting Items for Systematic Reviews and Meta-Analyses (PRISMA) flowchart.

This systematic review was conducted according to Preferred Reporting Items for Systematic Reviews and Meta-Analyses (PRISMA) guidelines using the MEDLINE database (PubMed) [39] (Figure 1). The following keywords were entered into the database: whole ovary, cryopreservation, transplantation. Out of 140 records, only peer-reviewed research articles focusing on the subject and written in English were taken into account for eligibility assessment (*n* = 69). Among studies performed on animal models, the following criteria led to the exclusion of 16 papers: (i) cryopreservation protocols for small animal models; (ii) transplantation of fresh whole ovaries; (iii) avascular transplantation. All studies using whole human ovaries were included, resulting in a total of 53 papers.

## 3. Challenges and Solutions

Although whole ovary cryopreservation and transplantation has yielded several live births in large animals [38,40,41], the technique involves a number of critical steps. The three main hurdles of whole ovary cryopreservation and transplantation are linked to (A) the surgical skills required to accomplish retrieval of a whole ovary with its vascular pedicle, (B) the cryopreservation technique adopted for freezing of the intact organ, and (C) successful execution of functional vascular reanastomosis upon thawing.

### 3.1. Whole Ovary Removal

In order to successfully harvest the entire ovary with its vascular pedicle for cryopreservation and future vascular reimplantation, great care must be taken when dissecting the ovarian vessels within the infundibulopelvic ligament. Indeed, the ovarian pedicle must be long enough (≥5 cm in humans) to allow processing of the graft and ensure perfect suture of donor vessels to recipient vessels of similar diameter [42,43]. Another crucial aspect is shortening the ischemic interval between ovary removal and cryopreservation as much as possible to avoid injury to ovarian cells [42,44,45,46]. For this reason, in the majority of studies, whole ovary removal is performed by laparotomy. However, some authors have demonstrated the feasibility of the procedure by laparoscopic surgery in sheep [47,48,49,50] and humans [42,51], despite the very twisted appearance of the ovarian vessels.

### 3.2. Freezing of a Whole Ovary

#### 3.2.1. Freezing Challenges

Developing a successful cryopreservation protocol for large organs like whole ovaries is extremely challenging [52]. Unfortunately, the success rate of freezing is inversely proportional to the complexity of the biological system being cryopreserved [53]. Numerous issues are associated with freezing and thawing of a whole ovary while preserving its viability upon thawing, including heat and mass transfer, poor heat transfer between the core and periphery of the organ, and adequate distribution of the cryoprotectant throughout the organ to prevent ice formation [54,55,56,57,58,59].

A preliminary in vitro study utilizing intact porcine ovaries demonstrated the beneficial effect of cryoprotectant use on the preservation of the ultrastructural integrity of primordial follicles [60]. Two further studies conducted on sheep ovaries underlined the protective impact of adding cryoprotectant to the freezing medium, compared to using a simple freezing medium composed of Ringer’s solution without any cryoprotective agents [61,62]. Wallin et al. used propanediol [61], while Milenkovic et al. opted for dimethyl sulfoxide (DMSO) as cryoprotectants [62]. Viability was assessed the same way in both studies and the results were similar. Briefly, the whole ovary was placed in a perfusion tray after thawing and stimulated with an adenylate cyclase stimulator (forskolin). Cyclic adenosine monophosphate (cAMP) and hormone levels were measured after stimulation and no significant difference was identified between groups with or without cryoprotectant. After perfusion, cells were isolated and cultured. Addition of human chorionic gonadotropin (hCG) to cell cultures resulted in higher progesterone levels in the group cryopreserved with cryoprotectant than the group cryopreserved without cryoprotectant. Histological assessment showed well-preserved tissue architecture, with the presence of small follicles in all groups immediately after thawing. However, the ovarian architecture of ovaries frozen without cryoprotectant became disrupted, with signs of edema, disorganized stroma and shrunken follicles after perfusion, emphasizing the benefits of adding cryoprotectant to the freezing medium.

Optimal distribution of cryoprotectant throughout the organ and its vascular network is of paramount importance to avoid ice formation in intracellular and extracellular locations, particularly in blood vessels [55,63,64,65]. Intravascular ice formation irreversibly disrupts the endothelial cell layer, resulting in thrombotic events after transplantation of a frozen–thawed whole ovary [55,63,65,66,67,68]. Indeed, the large volume and complexity of a whole organ somewhat hamper cryoprotectant diffusion, making it difficult to obtain even distribution within the organ without inducing unacceptable levels of toxicity caused by high doses of cryoprotectant [56,69,70,71,72,73]. Gerritse et al. stressed the importance of associating perfusion of the ovarian pedicle with submersion of the bovine ovary using a cryoprotective solution to confer the highest degree of protection [74]. Another group investigated whether in vivo perfusion of the ovarian artery before removal of the ovine ovary could achieve optimal cryoprotectant distribution throughout the organ but found that only 10% of the ovarian tissue was actually perfused. They concluded that perfusion should be performed in vitro, as described above [75].

Various studies have demonstrated a deleterious effect of both perfusion and cryopreservation on the ovarian vasculature, especially on the microvasculature within the ovarian medulla [55,61,71,76,77,78]. This could lead to capillary microthrombi arising from the ovarian medulla, remote from the anastomotic site, causing local ischemia. This process might be responsible for the poor follicle outcomes reported in several animal studies after vascular transplantation of whole ovaries, despite maintaining vascular patency of the anastomotic site [40,68,78]. An in vitro study performed with intact sheep ovaries demonstrated that cryopreservation significantly increased endothelial cell disruption and smooth muscle damage, but that adding the antiapoptotic agent sphingosine-1-phosphate to the cryopreservation medium (composed of 1.5 M DMSO) did not improve ovarian or vascular tissue viability following cryopreservation [79]. The authors concluded that cell loss was either due to necrosis caused by intracellular ice formation and osmotic stress, or that they might have targeted the wrong apoptotic pathway.

To further confirm that perfusion alone and perfusion associated with freezing have deleterious effects on endothelial cell function compared to unperfused fresh controls, the same team studied a number of endothelial cell-related genes in the medulla and the pedicle [78]. Like expression of endothelial nitric oxide synthase, cryopreservation did not affect expression of endothelin-1 in the pedicle, but it was significantly downregulated in the medulla compared to perfusion alone. This suggests that small medullary vessels are more sensitive to the effects of cryopreservation, limiting their reactivity to vascular tone. On the other hand, expression of endothelin-2 was significantly upregulated in the pedicle after perfusion, suggesting that hypoxic conditions may have been created there during perfusion. However, since hypoxia stimulates endothelins, including endothelin-1 and -2, the authors could not explain why endothelin-1 did not follow the same pattern. Regarding cellular apoptosis, upregulation of proapoptotic Bcl-2-associated X protein (BAX) expression and downregulation of antiapoptotic Bcl-2 expression in response to perfusion and freezing suggested detrimental effects of both of these processes on cell survival. Interestingly, caspase-6 expression in the medulla was downregulated after perfusion, indicating that final execution of cell death may be suppressed within the medulla, despite potential upregulation of the apoptotic pathway by BAX, but the reason for this remains unknown. Expression of thrombospondin-1 was found to be downregulated in the medulla and pedicle after perfusion. According to the authors, downregulation of thrombospondin-1 expression may imply that wound repair mechanisms are recruited due to perfusion. They also concluded that both perfusion alone and in combination with freezing had a deleterious impact on endothelial cell function [78].

#### 3.2.2. Freezing Solutions

Considering all the issues involved in cryopreserving a whole organ, animal models used to investigate freezing protocols should have ovaries similar to humans in terms of size, architecture and ovulation patterns, with comparable follicle distribution within the cortex [72]. Porcine, ovine and bovine ovaries may be considered appropriate models [72,80], whose characteristics are summarized in Table 1. Three different freezing techniques have been developed over time: slow freezing, vitrification and directional freezing. The two main freezing procedures used for whole ovary cryopreservation are slow freezing and vitrification, but directional freezing is gaining ground thanks to recent encouraging results. The following paragraphs will look at the three different ways of cryopreserving a whole ovary, with their advantages/disadvantages summarized in Table 2.

##### Slow freezing

The purpose of slow freezing is to achieve a freezing rate that is slow enough to dehydrate cells and avoid intracellular ice crystallization, but fast enough to prevent cellular osmotic stress. Martinez-Madrid et al. developed a slow-freezing protocol for human ovaries [81]. Three human ovaries were perfused and bathed for 5 min in 10% DMSO at a flow rate of 2.5 mL/min and further pre-equilibrated for 10 min in the same solution. Although follicle viability, as assessed by vital fluorescent staining, was significantly decreased in frozen–thawed ovaries (75.1%) compared to fresh ovaries (99.4%), cryoinjury was minimal. Only 2.4% of follicles were moderately damaged, and no dead follicles at all were encountered immediately after thawing. Apoptosis and ultrastructure were assessed in a follow-up study [82], but the authors did not observe any post-thaw apoptosis in primordial/primary follicles, nor in vessels or stromal cells. Furthermore, no ultrastructural alterations were seen in any cell type, which would indicate that human ovarian vessels are fairly resistant to cryopreservation, at least in the case of slow freezing [82].

However, immediate post-thaw viability assessment might be a poor predictor of long-term ovarian survival after whole ovary cryopreservation [68,79]. For this reason, several teams have developed functional in vitro assays to try to identify optimal freezing conditions to fully protect all ovarian compartments in large animal models. They include: (i) measuring the release or secretion of specific proteins like sex hormones [62,83], lactate dehydrogenase [38,84], and cAMP [61,62]; (ii) evaluating cell proliferation through bromodeoxyuridine uptake [79]; (iii) measuring glucose consumption and lactate release [74]; (iv) conducting combined assessment of glucose uptake and histology through tests like the methyl thiazolyl tetrazolium (MTT) assay [66]. An in vitro study performed using intact sheep ovaries showed good evidence of survival and restored proliferation in slow-frozen whole ovaries compared to fresh controls using a wide range of functional assays [79]. Sheep ovaries were perfused and bathed for 60 min at a flow rate of 0.5 mL/min in a freezing medium composed of 1.5 M DMSO. Granulosa cell viability was maintained after cryopreservation with respect to cell membrane integrity and metabolic activity. Cell proliferation was assessed by Ki67 expression and bromodeoxyuridine uptake following culture of ovarian cortical strips, and the findings were similar between fresh and frozen–thawed tissue. In addition, viability staining with 5(6)-carboxyfluorescein-diacetate succinimidyl-ester (CFDA SE) indicated the presence of metabolically active primordial and primary follicles in cryopreserved tissue, and viable follicle density also appeared to be unaffected by the cryopreservation process. This study complemented previous research findings and confirmed that such functional assessments of ovarian cell viability show similar results between fresh and slow-frozen whole ovine ovaries [79].

Some teams have demonstrated that perfusion for as long as 60 min is required to ensure optimal preservation of the different layers of intact ruminant ovaries [38,46]. Westphal et al. investigated the optimal cryopreservation protocol for whole ovaries in a bovine model [46]. They found that perfusion/submersion of whole bovine ovaries for 60 min at a flow rate of 2.5 mL/min using DMSO as a cryoprotectant was superior to other tested protocols, which varied in perfusion duration and type of cryoprotectant. They subsequently applied their optimized protocol to human ovaries and obtained over 95% protection against cryodamage on all tissue levels [46]. A cooling rate of 0.5 °C/min and seeding temperature of −5 °C were shown to be appropriate for slow freezing of cow ovaries [85].

Interestingly, one team attempted to freeze whole sheep ovaries using a DMSO-free cryoprotectant solution based on trehalose [86]. They obtained satisfactory results in terms of follicle survival rates and stromal apoptosis, but further studies need to be initiated to assess the functional recovery and endothelial viability of frozen–thawed whole ovaries with trehalose as a cryoprotectant.

It should be noted that all live births reported after whole ovary cryopreservation and transplantation to date were obtained using the slow-freezing procedure [38,40,41].

##### Vitrification

The principle of vitrification is to prevent ice formation during ultra-rapid cooling by use of highly concentrated cryoprotective solutions aiming to increase tissue fluid viscosity, which in turn produces an amorphous state of the liquid, without any crystallization [87,88]. To our knowledge, no attempt has yet been made to vitrify an entire human ovary, but the technique has been scrutinized in a series of papers on whole ovary freezing in sheep. An initial study on ovine ovaries by Courbière et al. compared two vitrification solutions described by Fahy’s team named VS1 (DMSO, acetamide, propylene glycol and polyethylene glycol) and VS4 (DMSO, formamide and propylene glycol) [69,77]. Perfusion was performed at a rate of 0.35 mL/min, with a stepwise increase in concentrations of cryoprotectant. From a histological perspective, 53.5% ± 3.2% (mean ± SEM) of follicles remained normal with VS4, compared to 25.2 ± 7.0% with VS1. VS4-treated ovaries showed more cytoplasmic anomalies, but VS1 appeared to induce more nuclear and combined anomalies. The authors concluded that the VS4 cryoprotectant was superior to VS1. However, it should be pointed out that the vascular pedicle was fractured in most samples during the rapid rewarming procedure, although there was no mention of reperfusion difficulties. The same team identified a major issue in a follow-up study, namely the challenge of thawing a vitrified ovary quickly enough to prevent ice formation [70]. It was demonstrated that VS4 does not impregnate sheep ovarian cortex and the pedicle sufficiently with cryoprotective agents, as illustrated by differential scanning calorimetry and MTT assays [71,89]. They then attempted vascular grafting of vitrified ovine ovaries using VS4, but outcomes were alarming, with most of the ovaries found to be atrophic at degrafting about one year later [67] (see “Vascular transplantation solutions”). This team finally concluded that VS4 may not be the most suitable vitrification solution.

Another group studied vitrification of whole goat ovaries using a solution containing DMSO and ethylene glycol [90]. They noted that follicle survival after two days of culture was only 18.4% ± 0.8% in the vitrified-warmed group compared to 55.1% ± 2.1% in the fresh tissue group. This was confirmed by ultrastructural analysis, which showed an escalation in follicle degeneration in the frozen group [90].

Vitrification of whole cow ovaries was also investigated using a vitrification solution containing DMSO and propylene glycol [91]. The ovaries were randomly divided into different groups according to perfusion pressure and perfusion duration. It was established that a perfusion pressure of 100 mmHg and perfusion duration of 40 min were appropriate for vitrification of cow ovaries in terms of follicle viability, proportion of morphologically normal primordial follicles and hormone levels in culture supernatants [91].

A comparative study of thawing protocols was then performed using vitrified cow ovaries [92]. The best thawing protocol would ideally prevent recrystallization by applying a sufficiently rapid thawing rate without inducing any osmotic stress in cells due to abrupt melting of the extracellular solution [93]. Frozen ovaries were either directly plunged into a water bath of variable temperature (37 °C, 39 °C, or 41 °C) for 3 min or thawed in two steps, first exposed to air for 30 s at room temperature, before being submerged in the water bath. They were then perfused through the ovarian artery with pre-warmed medium (37 °C) supplemented with decreasing sucrose concentrations for 30 min in order to remove the cryoprotectant, as routinely practiced. The authors found that the two-step thawing procedure using a water bath at 39 °C resulted in a significantly greater percentage of morphologically normal primordial follicles and higher hormone levels in culture supernatants. This was in line with the ultrastructural analysis of oocytes, which showed fewer alterations and smaller average vacuole size in the ooplasm with the latter rewarming protocol [92].

Few studies have compared the efficiency of vitrification and slow-freezing procedures for whole ovary cryopreservation. Vitrification appeared to be more appropriate for freezing of cow ovaries [84]. The viability of isolated follicles, evaluated using trypan blue, was significantly higher in the fresh group (84.5% ± 2.6%) than in the vitrified group (63.7% ± 4.3%), which in turn was significantly higher than in the slow-frozen group (51.9% ± 3.5%) (*p* < 0.05). This was confirmed by histological examination of morphologically normal follicles and functional assessment, which was conducted by sampling hormone levels after 14 days of culture of ovarian biopsies. Concerning sheep ovaries, a study compared post-grafting outcomes of both vitrified and slow-frozen whole ovaries [41]. According to the authors, vitrification appeared to be slightly superior to slow freezing, although unsatisfactory follicle outcomes were obtained in both groups and the only live birth in the study occurred in the slow-freezing group.

##### Directional freezing

Directional freezing is an emerging freezing technique based on a thermodynamic principle, whereby tissue is moved through a preprogrammed temperature gradient at a speed that determines the cooling rate. It confers a precise and uniform cooling rate because the freezing front moves from one side to the other. This strategy allows cooling of the sample as ice crystals grow in the opposite direction to the tissue movement [57,94]. Arav et al. detected similar follicle survival rates between fresh (99.7 ± 0.7%) and directional frozen–thawed whole sheep ovaries (97.7 ± 3.1%) using live/dead fluorescent stains [95]. This was further confirmed by histological assessment of follicle morphology, which was well preserved after thawing. Another study in the same animal model demonstrated that morphological parameters and hormone secretion levels of cultured whole ovaries frozen–thawed by directional freezing were not different from fresh controls [96]. These results support previous findings by the same team, since they indicate that primordial follicles are not the only ones to remain functional after being subjected to directional freezing of whole ovaries [97,98], but that larger steroid-producing follicles are similarly unaffected by the freezing procedure and interaction with DMSO.

Regarding application of this technique to freezing of whole human ovaries, Patrizio et al. successfully cryopreserved 11 ovaries from premenopausal women using directional freezing [99,100], with their contralateral ovary serving as fresh controls. Histological analysis revealed a similar morphology in follicles, cortical stromal cells, small vessels and the vascular pedicle between thawed and control ovaries. Follicle counts were also comparable. While not statistically significant, an increase in apoptotic markers, analyzed by immunohistochemistry and western blot, was noted in frozen–thawed specimens [100].

Another study compared directional freezing with slow freezing of whole sheep ovaries, using fresh ovaries as controls [97]. The proportion of morphologically normal follicles identified by histology immediately after thawing in the directional-frozen group (89% ± 1.7%; mean ± SEM) was significantly higher (*p* < 0.05) than in the slow-frozen group (61% ± 3.4%) and similar (*p* = 0.75) to the fresh control group (96% ± 1.5%). Functional evaluation of biopsies cultured in vitro revealed that a comparable number of primordial follicles grew to the primary stage after seven days of culture in directional-frozen ovaries and fresh controls, whereas slow-frozen fragments showed hampered developmental competence. However, these results must be viewed with caution. Indeed, the very low numbers of morphologically normal follicles retrieved immediately after thawing of slow-frozen ovine ovaries is inconsistent with results obtained in other studies investigating the efficiency of slow freezing in the same animal model. Onions et al. found the density of metabolically active primordial and primary follicles to be similar to fresh controls after slow freezing [79]. This discrepancy may be explained by the different slow-freezing protocols used to cryopreserve ovine ovaries, the biggest difference residing in perfusion duration, with Maffei’s protocol actually 12 times shorter than that of Onions (5 min vs. 60 min, respectively) [79,97]. Complementary studies have also highlighted the importance of long perfusion times to allow optimal penetration of cryoprotectant before freezing of ruminant ovaries [38,46]. Hence, the slow-freezing protocol applied by Maffei’s team does not appear to be adequate to freeze whole ovine ovaries, so their conclusions may well lack validity.

It was further suggested that compared to slow freezing, directional freezing improved the viability of cryopreserved ovarian tissue, not only when used on whole organs, but also ovarian fragments. Moreover, whole ovaries were better preserved than ovarian tissue specimens [98]. Nevertheless, these results are similarly open to question, as above.

Maffei et al.’s assumptions that directional freezing of whole sheep ovaries is superior to slow freezing and that structural and functional outcomes are similar to fresh controls were further undermined by a study on porcine ovaries [101]. The authors of the latter found that the two cryopreserved groups were not significantly different from each other. Importantly, histological evaluation demonstrated an overall decrease in follicle counts in both slow-frozen (46%) and directional-frozen (50%) ovaries compared to fresh controls (*p* < 0.05), in line with live/dead viability assays (60% and 58% in directional freezing and slow freezing vs. 74% in fresh). Here too, caution must be exercised when interpreting and comparing the results, because both freezing protocols differed from Maffei’s experiments and the animal model was different. However, regarding apoptosis assays, slightly better results were obtained with directional freezing, leading the authors to conclude that further investigations are needed into this cryopreservation technique.

Vascular transplantation of directional-frozen whole ovaries was also attempted in sheep, and signs of ovarian function were documented for up to 6 years [102] (see “Vascular transplantation solutions”).

### 3.3. Vascular Transplantation of a Whole Ovary

#### 3.3.1. Vascular Transplantation Challenges

Vascular transplantation of fresh whole ovaries has been largely successful in animal models (rats [103,104,105], rabbits [106,107,108,109,110,111,112,113,114], sheep [47,49,67,68,78,115,116,117,118,119,120,121], pigs [122,123], dogs [124] and monkeys [125,126]). Encouraging results have also been reported on transplantation of frozen–thawed whole ovaries in rats [66,127,128,129], rabbits [130] and sheep [38,40,41,48,49,50,67,68,78,95,102,131], which will be detailed further in this review. In humans, however, only fresh whole ovary transplantation has been undertaken so far [51,132,133,134], yielding one live birth to date [51].

For a successful outcome, vascular reanastomosis needs to be carried out by an experienced surgical team, including specialists in microvascular surgery, due to the level of complexity. Indeed, the reported diameter of the human ovarian artery is around 1 mm, and the ovarian vein approximately 1.2 mm [43]. Although the ovarian vein is slightly larger, its anastomosis may be more problematic because of its thin walls and poorly defined lumen [64].

Furthermore, selection of recipient vessels must be carefully considered to keep any vessel size discrepancy to a minimum, as sudden changes in vessel caliber cause turbulence to blood flow, which predisposes them to thrombosis [43,135,136]. Thrombosis is indeed the most common cause of anastomotic failure and is regulated by Virchow’s triad (damaged endothelial lining, hypercoagulability, turbulent blood flow). End-to-end vascular anastomosis is the most reliable approach, with an accuracy of 96%, and should be implemented when the vessel mismatch ratio is below 1: 1.5 [43,135]. If the vessel mismatch ratio is above 1: 1.5, a number of microsurgical techniques may be applied to manage donor versus recipient vessel discrepancy, such as oblique cutting, fish-mouth cutting, the sleeve technique or end-to-side anastomosis [43,135,137]. The goal is to achieve optimal blood flow through the connection and reduce the risk of thrombosis. In addition, sutureless methods like adhesives, laser welding or stents may also be considered depending on the surgeon’s experience [135,138,139]. Unfortunately, there is no consensus to date on the ideal technique to resolve the problem of vessel size mismatch [43,135,137]. Moreover, these complex anastomoses often lead to higher rates of complications, so the choice of a particular technique has to be made according to each individual case [43,135,137].

#### 3.3.2. Vascular Transplantation Solutions

This section specifically focuses on reviewing vascular transplantation of frozen–thawed whole ovaries in animals (Table 3), a technique only performed in rodents and sheep so far.

##### Rodents

Wang et al. reported the first ever case of restored fertility after vascular transplantation of cryopreserved whole ovaries [127]. The authors used female adult donor rats and removed the right ovary as well as the fallopian tubes and upper segment of the uterus en bloc, along with an aorta-vena cava segment, including the branches of the right ovarian vessels. Seven specimens were cryopreserved according to the slow-freezing technique after being heparinized and perfused for 30 min at 0.35 mL/min with increasing concentrations of DMSO (0–1.5 M), and then stored in liquid nitrogen. Upon rapid thawing, syngeneic recipient rats were heparinized and both ovaries, fallopian tubes, and the upper third of the uterus were removed. Transplantation was performed using the same technique as previously described for fresh samples [104]. In brief, the recipient’s aorta and vena cava below the left renal artery were prepared and flushed with heparinized Ringer’s solution for end-to-side aorta-aortic and veno-venous anastomosis using continuous suture. The uterus, meanwhile, was joined to the corresponding host segment. Four of the seven (57%) cryopreserved transplants showed resumption of ovarian function for up to 60 days, but only one ongoing pregnancy (14.3%) was identified and no live birth was recorded. Interestingly, tubal and uterine morphology were indistinguishable from non-operated controls [127]. A more comprehensive analysis of this experiment was published later, in which the authors pointed out that serum follicle-stimulating hormone (FSH) values were higher and serum estradiol lower in operated animals than in controls (*p* < 0.05), but not reaching castrated levels. They also noted fewer follicles in cryopreserved whole ovary transplants (*p* < 0.01), indicating a compromising effect of freezing on ovarian function [128].

In a follow-up study conducted by the same group, ovarian function resumed in eight out of ten (80%) animals that underwent vascular transplantation of cryopreserved whole ovaries, and spontaneous pregnancy occurred in two of them (20%) [66]. It was suggested that the lower serum estradiol levels seen after transplantation of frozen–thawed whole ovaries could be due to increased apoptosis in cryopreserved ovaries, as compared to fresh intact ovary transplants. This study confirmed the poor efficiency of the procedure and the authors concluded that improvements needed to be made in terms of surgical and freezing techniques [66].

Recently, the efficacy of whole ovary cryopreservation and transplantation was investigated in a POI rat model, simulating female patients with cancer treated by chemotherapy [129]. POI was induced in 25 recipient rats by administering cyclophosphamide and evidenced by rising serum FSH levels and decreasing progesterone and estradiol values. Ovaries from normal donor rats were harvested and cryopreserved according to the technique described by Wang et al. [127], but with a newly developed perfusion apparatus. Transplantation to recipient POI rats was performed in line with the methodology described earlier [127], and ovarian function and fertility were assessed for 8 months, after which the animals were euthanized. Fourteen of the 20 surviving POI rats (70%) recovered ovarian function 2 weeks postgrafting, with normal hormone levels restored 4 weeks after transplantation, persisting until the end of the study. Four of them (20%) became pregnant and delivered live offspring. Furthermore, second and third generations of rats were born from the initial offspring of the transplanted rats, indicating that the procedure did not affect viability or fertility in future generations. The fact that no pregnancy occurred in the ten remaining rats with restored ovarian function may be explained by adhesions around the transplantation site found at autopsy. Although the weight of the ovaries from the fourteen rats that recovered cyclicity was similar to that of sham-operated rats, the number of preantral follicles was significantly lower (*p* < 0.05). In the six animals that did not recover ovarian function, the grafted ovaries and vascular pedicle were atrophic or absent, with severe adhesions, based on postmortem examination. All in all, these results are encouraging and highlight the need for protocols to minimize postoperative inflammatory effects, adhesions, and thrombosis before human trials can commence [129].

Transplantation of cryopreserved whole ovaries has also been attempted once in rabbits [130]. In this study, bilateral oophorectomy was performed, with one ovary cryopreserved and the contralateral ovary fixed immediately to serve as a fresh control. The experimental ovary was harvested with its vascular pedicle dissected up to 2.5 cm from the ovarian hilum and cryopreserved as previously described in rats [127]. Autologous vascular transplantation was carried out to the groin pocket, with end-to-end vascular anastomosis to the inferior epigastric vessels (recipient) using interrupted 10–0 nylon stitches [130]. To avoid thrombotic events in the anastomotic site, lidocaine was applied topically to relieve vasospasm and vessels were flushed with heparinized Ringer’s solution. Anticoagulation therapy (heparin injections) was continued for seven consecutive days after grafting and one injection of urokinase was administered after surgery. Ten of the twelve rabbits (83.3%) recovered ovarian function, which was maintained throughout the total duration of the study (6 months). Mean follicle density was significantly lower at the 6-month post-transplantation follow-up than in control ovaries (*p* < 0.0001). This strongly suggests that some degree of ischemic damage is present even after successful vascular transplantation of cryopreserved whole ovaries, which may be due to cryoinjury to blood vessel walls during the freezing procedure. The two remaining rabbits that did not regain ovarian function showed fissured mesenteric fat adjacent to the ovary after thawing, which may also reflect unstable freezing conditions that injure the architecture of the graft and have a devastating impact on biological tissue [130].

##### Sheep

Sheep have been extensively used in research into vascular transplantation of cryopreserved whole ovaries and are considered a preclinical model. While the small size of the ovary of this species should be borne in mind (only one-sixth of the human ovary), the characteristics of ovine and human ovaries are similar, with a collagen-dense outer stroma containing the primordial follicle pool.

In the first study reporting vascular transplantation of cryopreserved whole ovine ovaries, intact sheep ovaries were compared with cortical strips. In both cases, freezing was achieved with a slow-freezing protocol using 1.5 M DMSO and the samples were grafted to the same anatomical location, close to the rectus muscle [48]. Removal of ovaries destined for whole organ cryopreservation was performed laparoscopically by dissecting the ovarian vessels as close as possible to their aortic emergence. Prior to cryopreservation, the grafts were perfused with heparinized Ringer’s solution. For autotransplantation of the frozen–thawed intact ovary, end-to-end microvascular anastomosis was performed with the inferior epigastric vessels of matching caliber using 8–10 interrupted sutures (9-0 or 10-0 Prolene), and heparin was topically administered. After surgery, the animals were anticoagulated using heparin for 3 days. Although immediate vascular patency through the anastomosis was 100%, only 27% (3/11) were patent 8–10 days after transplantation. A possible explanation for this early loss of vascular patency may be that cryopreservation induced injuries to the endothelial lining of the vessels, particularly at the anastomosis site, causing thrombotic events. In fact, low tolerance of vessels to cryopreservation was suggested by transmural necrosis observed in the walls of some vessels. Primordial follicle density was significantly higher in the patent anastomosis group than in the non-patent group, which was only 7% of the patent group (*p* = 0.001). Unfortunately, the authors did not investigate follicle density in the cortical strip group.

In a subsequent study, they compared different microvascular anastomosis techniques, evaluating success rates of vascular transplantation of both fresh and frozen–thawed whole ovaries using end-to-end, end-to-side or fish-mouth-modified end-to-end anastomosis to the inferior epigastric vessels [49]. Immediate vascular patency was achieved in all groups and preserved after 8–10 days of follow-up in more than 60% of cases (5/8) when end-to-end vascular anastomosis was used (2/2 in fresh tissue; 3/6 in frozen–thawed tissue). However, in the case of vessel size discrepancy, vascular patency at follow-up was much lower, falling to around 30% (2/6) after end-to-side anastomosis (1/2 in fresh tissue; 1/4 in frozen–thawed tissue) and 0% when the fish-mouth technique was applied (0/2 and 0/5, respectively). The authors also reported a significantly longer ischemia time when the fish-mouth technique was used compared with end-to-end (*p* < 0.01) or end-to-side (*p* = 0.05) anastomosis, which may have damaged endothelial cells and led to vessel occlusion. They finally concluded that end-to-end anastomosis should be carried out where feasible, and in the case of vascular discrepancy between donor and recipient vessels, end-to-side anastomosis is superior to fish-mouth incisions [49]. This study also raised another issue, namely the complexity of cryopreserving a whole ovary (see “Freezing challenges”).

The same group assessed long-term results of whole ovary cryopreservation and transplantation, using the same freezing and transplantation techniques as Bedaiwy et al. [48,50]. They found that only 25% (2/8) of frozen–thawed ovaries were functional 5 months after transplantation [50]. However, a fully functional vascular network was identified in these successful grafts based on immunohistochemical analysis showing expression of factor VIII, vascular endothelial growth factor (VEGF) and smooth muscle cell actin (SMCA). In addition, three cumulus oocyte complexes were retrieved from one ewe out of two that were responsive to FSH stimulation, but none fertilized.

Another group described a different microsurgical approach and used directional freezing for whole ovary cryopreservation [131]. The frozen–thawed ovary was transplanted by end-to-end anastomosis to the freshly dissected vascular pedicle of the contralateral ovary. The time needed to perform these complex anastomotic procedures was about 60 min and 40 min for the arterial and venous anastomoses, respectively. It must be borne in mind that a prolonged warm ischemia time, which was close to 2 h, may cause major damage to tissue. To keep this damage to a minimum, it is advisable to cool the ovary during the microsurgical process, which is in line with clinical routines in solid organ transplantation [140]. In order to minimize the risk of thrombotic events, the ovaries were flushed with heparin upon thawing, and hyaluronic acid gel was applied to the grafts to reduce development of adhesions, which are an impediment to pregnancy. Successful anastomosis was observed in 62.5% (5/8) of animals, but only 37.5% (3/8) recovered progesterone cyclicity around 5–9 months after transplantation [131]. A follow-up study reported the aspiration of six oocytes from two of the three cycling sheep [95]. Embryo development up to the 8-cell stage occurred after parthenogenetic activation. At one year of follow-up, magnetic resonance imaging confirmed normal ovarian size and vasculature. These two sheep continued to show normal hormone cyclicity for up to three years after transplantation [95]. Furthermore, six years later, histological evaluation of these two grafts revealed normal tissue architecture, intact blood vessels and follicles at various stages [102]. Hormone cyclicity was even evidenced in one of the animals [102]. This is currently the longest documented duration of ovarian function after whole ovary cryopreservation and transplantation.

In 2006, Imhof et al. reported the first live birth after transplantation of a cryopreserved whole ovary in a large animal model [40]. However, they also noted that follicle survival after 18–19 months was only 1.7% to 7.6% of that in non-transplanted control ovaries, indicating that further improvements to the technique were required. To this end, another group developed an additional surgical technique [68]. The ovarian vein was cut at its emergence from the vena cava and an aortic patch containing the origin of the ovarian artery was taken along with the ovary. The specimen was subsequently heparinized and cryopreserved using a slow-freezing procedure in 1.5 M DMSO. Once thawed, the ovary was transplanted to the neck and anastomosis was performed end-to-side to the carotid and jugular vessels. Only three out of seven (42.9%) grafted animals recovered cyclicity, although immediate vascular patency was evidenced in 87.5% (7/8) and maintained for more than 7 months. Very severe follicle loss (>90%) was nevertheless identified soon thereafter (*p* < 0.001). Surprisingly, the same loss was observed after transplantation of fresh ovaries that had been perfused with cold media and heparinized prior to grafting. The authors suggested that factors associated with cannulation and perfusion may have contributed to this depletion [68] (as described in “Freezing challenges”). Courbière et al. observed the same dramatic follicle loss after orthotopic grafting of both fresh and vitrified-warmed whole sheep ovaries [67].

In the two studies described above [68,69], fresh ovarian grafts were perfused during preparation of the grafting site. In order to assess the effect of perfusion more closely, a follow-up study compared 7-day transplantation of slow-frozen whole ovaries to that of freshly perfused and fresh whole ovaries [78]. For this study, the authors applied the slow-freezing procedure described earlier by Onions et al. [79] (see “Slow freezing”), and grafting was performed to the freshly dissected contralateral ovarian pedicle. Seven days after transplantation, vascular patency was evidenced in both fresh controls (4/4) and freshly perfused (4/4) ovarian transplants, while blood flow had ceased in 3/4 ewes transplanted with cryopreserved ovaries, with blood clots seen in the ovarian artery and ovarian medulla. Unfortunately, the only patent frozen–thawed ovarian transplant clotted during degrafting surgery. Prior to degrafting, the authors had perfused patent ovarian arteries with fluorescent microspheres. They observed extravasation in around 50% of blood vessels in freshly perfused ovaries, but no leakage at all was detected in non-perfused fresh whole ovaries, indicating a deleterious effect of perfusion on the endothelial cell lining. Regarding primordial/transitional follicle counts, although a considerable fall was encountered after transplantation of fresh and freshly perfused ovaries, these differences were not significant and were similar in both groups. However, significant primordial/transitional follicle loss (96%) was noted after transplantation of cryopreserved whole ovaries compared to their day 0 controls. Furthermore, postgrafting primordial/transitional follicle density was significantly lower in the cryopreserved group than in the fresh group. According to the authors, the fact that follicle loss in fresh and freshly perfused transplanted ovaries was not significant, whereas a significant decline was observed in cryopreserved ovarian transplants, suggests a freeze–thawing-mediated impact on follicle survival. Stromal cell proliferation and apoptosis, evaluated by immunohistochemistry using Ki67 and caspase-3 markers, was not significantly different between the three groups. However, a significant increase in cell proliferation and apoptosis was observed after transplantation of cryopreserved ovaries compared to their day 0 controls, suggesting an ongoing tissue regeneration process in transplanted frozen–thawed ovaries [78]. The authors then identified a series of endothelial cell-related genes whose expression was strongly affected by both cryopreservation and perfusion, supporting their previous assumption that both these interventions have deleterious effects on endothelial cell function (see “Freezing challenges”). They finally recommended that more specific anti-thrombotic agents be added to heparin to prevent platelet activation and clotting within the ovarian vasculature.

A later study investigated whether post-thaw perfusion of survival factors (angiogenic, antioxidant, antiapoptotic agents) and aspirin treatment could have beneficial effects on the restoration of ovarian function 3 months after transplantation of slow-frozen whole ovaries [38]. Survival factor supplementation yielded no benefits, but administration of preoperative aspirin resulted in higher rates of ovarian function resumption. Nevertheless, follicle survival of only 20% was identified within grafts showing restored function. In the second part of the study, the authors investigated the association of preoperative aspirin with additional postoperative anticoagulation regimens, including combinations of aspirin and enoxaparin, or aspirin and eptifibatide. Surprisingly, they obtained 100% (14/14) restoration of ovarian function, with high rates of natural fertility (pregnancy rate: 64% (9/14); live birth rate: 29% (4/14)).

At degrafting 11 to 23 months later, histological examination revealed a 60–70% follicle survival rate. This huge improvement in success rates of whole ovary cryopreservation and transplantation resulted from use of an optimal slow-freezing protocol for ovine ovaries (DMSO 1.5 M perfused for 60 min) and anti-thrombotic agents to prevent postoperative clotting. This was further emphasized by Torre et al., who transplanted both vitrified and slow-frozen whole ovaries to sheep (see “Vitrification”), but without anti-thrombotic therapy [41]. Although one live birth occurred in the slow-frozen group, resulting follicle survival rates were very poor in both groups at degrafting three years later (<99%), and comparable to those reported previously by other teams [40,67]. These results confirm the importance of applying adequate thrombosis prevention therapy. However, before whole ovary cryopreservation and transplantation may be implemented in clinical practice, researchers need to find the ideal cryoprotectant perfusion time for human ovaries of different sizes, optimize the duration and degree of anticoagulant treatment, and confirm whether offspring derived from the procedure are normal and healthy.

## 4. What Has Been Done in Humans So Far?

### 4.1. Fresh Whole Ovary Transplantation

To date, only a limited number of teams have attempted the exhausting and technically challenging feat of vascular transplantation of fresh whole ovaries in humans [51,132,133,134]. However, no attempts have yet been made to transplant a cryopreserved whole ovary in humans.

The first grafts of fresh whole ovaries were performed to the upper arm of the patient, and two teams reported successful heterotopic transplantation before initiating sterilizing pelvic irradiation [132,133]. In the first report, the patient was suffering from Hodgkin’s lymphoma. A cavity was created in her forearm by inserting a testicular prosthesis 2 months prior to transplantation in order to prepare the grafting site [132]. The ovary was then transplanted, and the ovarian vessels were branched end-to-end to the vessels from the humeral vascular bundle. The authors reported no disruption to the menstrual cycle, which remained regular for up to two years, with evidence of follicle growth in the transplanted ovary [132]. In the second report, the patient was suffering from cervical carcinoma and heterotopic autotransplantation of the ovary to the upper arm was performed in the context of radical hysterectomy prior to pelvic radiotherapy [133]. For over a year, there was evidence of regular menstrual cycles and follicle growth upon clinical examination and ultrasound. Unfortunately, local disease recurred one year later, and no long-term follow-up was reported.

A third team achieved the first successful orthotopic transplantation in a patient with Turner syndrome [134]. The transplant was retrieved from an immunologically matched donor sister through a Pfannenstiel incision. The ovarian vein was anastomosed end-to-side to the external iliac vein, and the ovarian artery was sutured end-to-end to the inferior epigastric artery by laparotomy. The ovary was fixed in its orthotopic position. Ovarian function resumed and was maintained for at least 2.5 years (follow-up duration). The patient also developed secondary sexual characteristics.

The last published study on fresh whole ovary transplantation was in 2008 between a pair of monozygotic twins discordant for POI [51]. The donor’s ovary was laparoscopically removed and transplanted to the recipient’s ovarian vessels through a minilaparotomy incision. This was actually the first report of a healthy live birth following orthotopic vascular transplantation of a fresh whole ovary in humans. These case reports provide proof of concept of the technical feasibility of the surgical procedure.

### 4.2. Whole ovary Removal with a View to Cryopreservation

Jadoul et al. demonstrated the feasibility of using a laparoscopic approach to harvest whole ovaries for further cryopreservation from nine patients [42]. This method maintains the minimally invasive nature of the procedure and should be the technique of choice for whole ovary cryopreservation. The authors stressed the importance of taking great care with dissection of the lumbo-ovarian ligament to ensure maximum length of the ovarian vessels (>5 cm), allowing dissection of the ovarian pedicle containing vessels of a suitable diameter (~1 mm), perfusion of the ovarian artery with cryoprotective solution, cryopreservation, and subsequent autotransplantation of the whole ovary. They also underlined the need to limit the interval between clamping of the ovarian pedicle and cryopreservation, in order to keep warm ischemia time as short as possible.

### 4.3. Cryopreservation of the Human Ovary

Few teams have had the opportunity to attempt cryopreservation of a whole human ovary (Table 4). Martinez-Madrid et al. were the first to report a successful slow-freezing protocol for human ovaries [81,82] (see “Slow freezing”). Although a significant decrease in the proportion of viable follicles (~30%) was observed after thawing, the authors did not detect any induction of apoptosis in any cell type, and no ultrastructural alterations were encountered.

Another team subsequently proposed a second protocol for slow freezing of the human ovary based on their results from cow ovaries [46] (see “Slow freezing”). In this protocol, the freezing medium used was the same, but the perfusion/submersion duration was 12 times longer than in Martinez-Madrid’s protocol. The authors obtained over 90% of morphologically normal follicles after thawing. Glucose uptake in cultured tissue fragments from cryopreserved ovaries reached 90–100% of that in fresh controls, and the endothelial lining appeared undamaged. However, no comparative study was conducted to identify the most adapted protocol.

Patrizio et al. were the first to describe successful cryopreservation of 11 human premenopausal ovaries using the directional freezing technique [99,100] (see “Directional freezing”). Their results were very encouraging, with no histological differences identified after thawing compared to fresh control ovaries.

The major issue when working with premenopausal whole human ovaries is their availability. In order to facilitate further research into development of an optimal freezing/thawing protocol, Milenkovic et al. proposed using postmenopausal ovaries [141]. Indeed, this would initially allow us to establish and improve cryopreservation protocols respecting the vasculature and stromal architecture of the human ovary, since postmenopausal ovaries are free of follicles. These selected protocols could then be applied to precious premenopausal ovaries in order to study their impact on follicle populations. Indeed, the first step to clinical application is development of an optimal freezing/thawing protocol for the human ovary. Once this goal is met, we may potentially move towards transplantation experiments performed by experts in microsurgery.

### 4.4. Recipient Pedicle Selection for Vascular Transplantation

Studies have investigated which recipient vessels should preferably be used to enhance the success rates of microvascular anastomosis. The choice of recipient pedicle should meet the following criteria [43,49]: (i) easy surgical accessibility of the pedicle; (ii) optimal vessel size match between recipient and donor ovarian vessels; (iii) possibility of postoperative ultrasound monitoring; (iv) easy oocyte pick-up from the transplant for in vitro fertilization. To enable orthotopic reimplantation (in an intraperitoneal grafting site) of the whole organ, the deep circumflex iliac vessels and deep inferior epigastric vessels have been proposed as potential candidates [49]. Based on anatomical evaluation of these two pedicles in 14 human female cadavers, the deep circumflex iliac vessels appeared to provide the best ovarian vessel size match to ensure reliable end-to-end vascular anastomosis. In fact, there was an optimal size match between these two pedicles in 13 out of 14 female cadavers [43]. The deep inferior epigastric vessels, on the other hand, are subject to variations. Their caliber varies along their course and their diameter gradually decreases until they enter the muscle, making them unreliable for successful anastomosis [43]. A freshly dissected contralateral ovarian pedicle may also be used, as described by Silber et al. [51], but the ovarian artery has a highly tortuous appearance [43]. When heterotopic transplantation is planned, both the cutaneous mammary branches and the antecubital vessels appear to fulfill the above-mentioned criteria [49].

## 5. Limitations

There are two inherent risks in whole ovary cryopreservation and transplantation, the first being the all-or-nothing nature of the procedure. Indeed, if any calamity occurs during the intervention, it results in loss of all the follicles present in the ovary, as opposed to use of cortical strips, where several attempts can be made with the remaining frozen fragments. The greatest risks in this instance are cryopreservation issues and clot formation during autotransplantation. Although Campbell et al. recently demonstrated a very effective freezing procedure and anti-thrombotic strategy in sheep [38], protocols still need to be adjusted to human beings.

The second crucial issue is the risk of malignant cell reimplantation [5,142,143]. While this threat also exists when transplanting frozen–thawed cortical strips, it is possible that the risk is higher when transplanting cryopreserved whole ovaries because the amount of grafted tissue is greater. An ovarian biopsy should therefore be frozen separately from the whole ovary in order to allow preimplantation analysis using extremely sensitive techniques and disease-specific markers, as carried out prior to cortical strip transplantation [144]. Among available techniques, histology and immunohistochemistry are sensitive enough to discern clusters of malignant cells, but isolated malignant cells may not be detected and potentially cause disease transmission upon transplantation [142,143,144]. Highly sensitive molecular biology techniques like RT-PCR are also used. They are able to identify small quantities of genetic material, whose sequences may correlate with known cancerous drifts. However, a positive result will only confirm the presence of malignant cells, but we still do not know how many malignant cells can actually cause disease recurrence [142,143,144]. Long-term xenografting experiments are nevertheless a valuable model to evaluate the risk of possible relapse. Xenotransplantation is usually performed in immunocompromised mice that serve as biological incubators, where potentially malignant cells within transplanted ovarian tissue are able to disseminate upon grafting. At degrafting, the transplants are subjected to the previously described sensitive tests and the animal is inspected for possible metastatic foci [142,143,144]. It is paramount for clinicians to bear in mind that ovarian biopsies used to perform all these tests cannot be transplanted to patients. Indeed, it is not possible to firmly rule out the presence of malignant cells in ovarian fragments that are transferred to patients.

If the risk of transmitting malignant cells is confirmed by preimplantation testing, one hypothetical option among others is use of the whole ovary as an ex vivo source of high-quality follicles [6,145]. Indeed, the whole ovary can be perfused in in vitro trays, and hormone stimulation and oocyte pick-up can then be performed ex vivo. In the early 1970s, researchers were able to maintain the premenopausal human ovary in perfusion for up to 8 h and induce ovulation 4 to 5 h after stimulation [146]. Later, rodent ovaries were perfused for up to 20 h, allowing detailed studies into ovulation mechanisms and gonadotropin responses [147,148,149,150,151,152,153,154,155,156]. However, these periods are considerably shorter than those required to retrieve a sufficient number of mature oocytes upon ovarian stimulation in order to achieve reasonable live birth rates [157]. Pig and cow ovaries can be perfused for up to 2 days while maintaining low cellular apoptosis rates during culture [158,159], and researchers were recently able to extend the duration of whole organ culture to 4 days using fresh and frozen–thawed intact sheep ovaries [96]. Of course, further developments are required to accomplish entirely ex vivo ovarian stimulation with a view to in vitro fertilization.

## 6. Conclusions

Whole ovary cryopreservation has been proposed as an alternative method that could extend the longevity of ovarian transplants, since the entire follicle pool would be transplanted and ischemic damage to the grafted ovary would be largely avoided thanks to vascular anastomosis. This review summarizes the challenges of this technique and examines various solutions proposed in the literature over the past 20 years.

Recent studies in the field have yielded very encouraging results and research efforts must be sustained [38,46,96]. As the permeability of any vascular anastomosis is critical to the outcome, teams investigating whole ovary transplantation should include experts with microsurgical skills. Furthermore, underlying mechanisms leading to follicle loss after transplantation of cryopreserved whole ovaries, even when vascular patency is maintained, need to be elucidated in order to develop innocuous freezing and grafting protocols. Finally, cryopreservation protocols must be adapted to human ovaries. Given their scant availability, initial studies could be conducted with postmenopausal ovaries, as proposed by Milenkovic et al. [141].

## Figures and Tables

**Table 1:** Ovarian characteristics in large animal models compared to the human ovary.

Species	Ovarian Volume (Mean ± SD)	Tissue Architecture Compared to the Human Ovary	Ovulation Pattern	Cycle Length (Days)
Human	6.5 ± 2.9 cm^3^ (Munn et al. 1986)	Not applicable	Mono-ovulatory	28–32
Cow	14.3 ± 5.7 cm^3^ (Gerritse et al. 2008)	Similar to human	Mono-, diovulatory	21
Pig	7.3 ± 2.2 cm^3^ (Gerritse et al. 2008)	Less fibrous	Multi-ovulatory	18–24
Sheep	1.0 ± 0.4 cm^3^ (Gerritse et al. 2008)	Similar to human	Mono-, triovulatory	16–17 (seasonal)

**Table 2:** Comparison of different freezing techniques.

	Advantages	Disadvantages
Slow freezing	Small amounts of cryoprotectant	Prone to intracellular ice formation Non-uniform cooling gradient between the core and periphery
Vitrification	No crystallization, amorphous state of liquids	Large amounts of cryoprotectant
Directional freezing	Controlled and uniform cooling gradient between the core and periphery	New technique, very few studies reported in the literature

**Table 3:** Transplantation of cryopreserved whole ovaries in animal models.

Species	Reference	Freezing Procedure	Transplantation	Follow-Up	Outcomes of WOCT
**Rodents**	Rat: Wang et al. 2002b, Yin et al. 2003	Slow freezing	Orthotopic, end-to-side (aorto-aortic and cava-caval)	≥60 days	∙ 4/7 (57%) resumed estrous cycles after 12 ± 2.5 days ∙ 1/7 (14.3%) conceived, but no live birth ∙ Hormones (baseline vs. postgrafting): FSH ↗ (21.3 ± 6.2 vs. 9.1 ± 0.4; *p* < 0.05); E_2_ ↘ (256.6 ± 61.8 vs. 416.9 ± 3.6; *p* < 0.05) ∙ Follicle counts (baseline vs. postgrafting): ↘ (*p* < 0.01)
	Rabbit: Chen et al. 2006	Slow freezing	Heterotopic (groin pocket), end-to-end (ovarian vessels to inferior epigastric vessels)	6 months	∙ 10/12 (83.3%) resumed ovarian function after 1 week ∙ No IVF attempts → no pregnancy ∙ Hormones (baseline vs. postgrafting): FSH ↗ (1.8 ± 0.5 vs. 15.5 ± 3.6; *p* < 0.01); P, E2 and LH = ∙ Follicle counts (baseline vs. postgrafting): ↘ (18.68 ± 3.86 vs. 13.99 ± 3.21; *p* < 0.0001)
	Rat: Qi et al. 2008	Slow freezing	Orthotopic, end-to-side (aorto-aortic and cava-caval)	≥42 days	∙ 8/10 (80%) resumed estrous cycles after 14 ± 3 days ∙ 2/10 (20%) conceived, but no live birth ∙ Hormones (baseline vs. postgrafting): E2 ↘ (258 6 98 pmol/l vs. 434 6 98 pmol/L) ∙ Follicle counts: not mentioned
	Rat: Ding et al. 2018b	Slow freezing	Orthotopic, end-to-side (aorto-aortic and cava-caval)	8 months	∙ 5/25 (20%) died from early postoperative complications (infection/anastomotic thrombosis) ∙ 14/20 (80%) resumed estrous cycles after 14 ± 3 days ∙ 4/20 (20%) conceived, yielding healthy offspring + second and third generations of rats from the initial offspring ∙ Hormones: FSH, P, E2, AMH = to sham-operated rats ∙ Follicle counts: ↘ (*p* < 0.05)
**Sheep**	Bedaiwy et al. 2003	Slow freezing	Heterotopic (rectus muscle), end-to-end (ovarian vessels to inferior epigastric vessels)	8–10 days	∙ 11/11 (100%) immediate vascular patency ∙ 3/11 (27%) maintained vascular patency after 8–10 days → 8/11 (73%) occluded vessels ∙ Hormones (baseline vs. postgrafting): ∙ Patent group: FSH = (182 ± 70.3 vs. 172 ± 42.0; *p* = 0.84); E2 = (166 ± 50.0 vs. 163 ± 32.6; *p* = 0.94) ∙ Non-patent group: FSH ↗ (103 ± 89.7 vs. 268 ± 109; *p* = 0.005); E2 = (199 ± 115 vs. 282 ± 132.0; *p* = 0.25) ∙ Follicle counts (patent vs. non-patent): ↘ (3.67 ± 2.08 vs. 0.250 ± 0.463; *p* = 0.001)
	Revel et al. 2004, Arav et al. 2005, 2010	Directional freezing	Orthotopic, end-to-end (ovarian vessels to contralateral ovarian vessels)	6 years	∙ 5/8 (62.5%) immediate vascular patency → 3/8 failure due to venous thrombosis (*n* = 1), torn artery (*n* = 1) and unknown reason (*n* = 1) ∙ 3/8 (37.5%) resumed P cyclicity 34 to 69 weeks after transplantation; 2/8 (25%): oocyte aspiration + embryo development up to the 8-cell stage ∙ 2/8 (25%) maintained ovarian function for up to 3 years and 1/8 (12.5%) for up to 6 years ∙ Hormones: not detailed ∙ Follicle counts: not detailed
	Imhof et al. 2006	Slow freezing	Orthotopic, end-to-end (ovarian vessels to contralateral ovarian vessels)	18–19 months	∙ 6/8 (75%) long-term vascular patency ∙ 4/8 (50%) resumed ovarian function 6 months after transplantation and P cyclicity resumed 12–14 months postgrafting ∙ 1/8 (12.5%) spontaneous pregnancy yielding healthy offspring ∙ Follicle survival rate: 1.7–7.6%
	Bedaiwy and Falcone, 2007	Slow freezing	Heterotopic (rectus muscle), ovarian vessels to inferior epigastric vessels	8–10 days	Successful vascular patency after 8–10 days: ∙ 5/8 with end-to-end anastomosis (WOCT = 3/6; fresh = 2/2) ∙ 2/6 with end-to-side anastomosis (WOCT = 1/4; fresh = 1/2) ∙ 0/7 with fish-mouth anastomosis (WOCT = 0/5; fresh = 0/2)
	Grazul-Bilska et al. 2008	Slow freezing	Heterotopic (rectus muscle), end-to-end (ovarian vessels to inferior epigastric vessels)	5 months	∙ 2/8 (25%) resumed ovarian function ∙ No information on maintained vascular patency ∙ 3 COCs obtained after FSH stimulation; 2 developed to metaphase II after in vitro maturation, but no fertilization ∙ Hormones: only 2 ewes in whose ovarian function was restored were considered, no statistical analysis ∙ Follicle counts: not mentioned
	Courbière et al. 2009	Vitrification	Orthotopic, end-to-end (ovarian vessels to contralateral ovarian vessels)	12 months	∙ 1/5 (20%) long-term vascular patency → 4/5 failure due to lumbo-ovarian pedicle thrombosis (*n* = 2), arterial thrombosis (*n* = 1) and pneumopathy (*n* = 1) (pedicle non-analyzable) ∙ 1/5 (20%) resumed ovarian function 6 months after transplantation ∙ Hormones: not detailed ∙ Follicle counts: total follicle loss
	Onions et al. 2009	Slow freezing	Heterotopic (neck), end-to-side (aortic patch to carotid artery and ovarian vein to jugular vein)	7 months	∙ 7/8 (87.5%) immediate and long-term vascular patency ∙ (In 1 case = freezing device malfunction → excluded from further analysis) ∙ 3/7 (42.9%) resumed ovarian cyclicity ∙ Follicles (pregrafting vs. postgrafting): ↘ (36.7 ± 5.7 vs. 2.3 ± 1.0; *p* < 0.05)
	Onions et al. 2013	Slow freezing	Orthotopic, end-to-end (ovarian vessels to contralateral ovarian vessels)	7 days	∙ 4/4 (100%) immediate vascular patency ∙ 1/4 (25%) vascular patency at degrafting → 3/4 (75%): arterial thrombosis + FMS extravasation in the medulla ∙ Follicle density (pregrafting vs. postgrafting): ↘ (0.42 vs. 0.02; *p* < 0.001)
	Campbell et al. 2014	Slow freezing	Orthotopic, end-to-end (ovarian vessels to contralateral ovarian vessels)	3 months	∙ 15/15 (100%) immediate vascular patency ∙ 4/15 (27%) restored ovarian function within 7 weeks of transplantation ∙ Follicle density within the functioning ovary (pregrafting vs. postgrafting): ↘ (2.48 ± 0.98 vs. 1.43 ± 0.10)
		Slow freezing	Orthotopic, end-to-side (ovarian vessels to uterine vessels)	11–23 months	∙ 14/14 (100%) immediate and long-term vascular patency ∙ 14/14 (100%) restored ovarian function within 3 weeks of transplantation ∙ 9/14 (64%) pregnancies + 4/14 (29%) and healthy live births. One offspring gave birth to second-generation lambs ∙ Follicle survival rate: 60–70%
	Torre et al. 2016	Slow freezing vs. Vitrification	Orthotopic, end-to-end (ovarian vessels to contralateral ovarian vessels)	3 years	∙ 6/6 (100%) immediate vascular patency with slow freezing vs. 67% (4/6) with vitrification ∙ (1 death occurred in both groups) ∙ 5/5 slow freezing + 6/6 vitrification (100%): restored hormone production ∙ 1/5 (20%) pregnancy and live birth of healthy offspring in the slow-frozen group. No pregnancy in the vitrified group ∙ Follicle survival rates (slow freezing vs. vitrification): 0.017% ± 0.019% vs. 0.3% ± 0.5% (*p* = 0.047)

Legend: WOCT: whole ovary cryopreservation and transplantation; E2: estradiol; FSH: follicle-stimulating hormone; IVF: in vitro fertilization; P: progesterone; LH: luteinizing hormone; AMH: anti-Müllerian hormone; COC: cumulus–oocyte complex; FMS: fluorescent microsphere.

**Table 4:** Cryopreservation protocols applied to human ovaries.

Reference	Number	Freezing Method	Investigated Outcomes
Martinez-Madrid et al. 2004, 2007	3	Slow freezing	Follicular, stromal and vascular viability, histological morphology, apoptosis, ultrastructural assessment
Patrizio et al. 2007, 2008	11	Directional freezing	Apoptosis, histological morphology
Milenkovic et al. 2011b	10 (postmenopausal ovaries)	Slow freezing	Histological morphology, ultrastructural assessment, androgen production during in vitro perfusion
Westphal et al. 2017	3	Slow freezing	Follicular and vascular viability, histological morphology, glucose uptake during tissue fragment culture
